# Diabetes Complications among Inpatients with Childhood and Young Adult–Onset Type 1 and 2 Diabetes

**DOI:** 10.1155/2024/9926090

**Published:** 2024-06-14

**Authors:** Kate Hawke, Soong Zheng Ng, Jessica Anderson, Raymond Dharmaputra, Prue Hogg, Angela Titmuss, Ashim Sinha, Anna McLean

**Affiliations:** ^1^ Diabetes and Endocrinology Department Cairns Hospital, 165 The Esplanade, Cairns, QLD 4870, Australia; ^2^ Logan Endocrine and Diabetes Services Logan Hospital, Loganlea Road, Meadowbrook, QLD 4131, Australia; ^3^ Diabetes and Endocrinology Department Gold Coast Health Service, 1 Hospital Boulevard, Southport, QLD 4214, Australia; ^4^ Women's and Newborn Services Royal Brisbane and Women's Hospital, Butterfield Street, Herston, QLD 4006, Australia; ^5^ Wellbeing and Preventable Chronic Diseases Division Menzies School of Health Research Charles Darwin University, Building 58 Royal Darwin Hospital Campus, Tiwi, NT 0810, Australia; ^6^ Department of Paediatrics Division of Women, Children and Youth Darwin Hospital, 105 Rocklands Dr, Tiwi, NT 0810, Australia; ^7^ College of Medicine and Dentistry James Cook University, McGregor Road, Smithfield, QLD 4878, Australia

## Abstract

**Aims:**

To assess morbidity among young people with diabetes presenting to a regional hospital in Northern Australia and compare the risk of complications among those living with type 2 diabetes (T2D) versus type 1 diabetes (T1D).

**Materials and Methods:**

A cross-sectional study of young people with T1D or T2D (diagnosed age 1–25 years) presenting to a regional Northern Australian hospital with any condition from 2015 to 2019. Demographics, cardiometabolic comorbidities, and diabetes-related complications were collected from individual medical records and compared between those with T1D and T2D.

**Results:**

Among 357 young people (192 had T2D, 165 T1D), the mean age was 22 years, the mean duration of diabetes was 6.7 years, 52% were Aboriginal or Torres Strait Islander, and 28% lived remotely. Cardiometabolic comorbidities (obesity, hypertension, and dyslipidaemia) and diabetes-related complications (microalbuminuria, amputation, and elevated non-alcoholic fatty liver disease score) were more prevalent in those with T2D compared to T1D, despite shorter disease duration and lower median HbA1c. When adjusted for age, sex, and BMI, the odds ratio (95% CI) for microalbuminuria was 4.8 (1.83–12.8) with T2D compared to T1D.

**Conclusion:**

In a cohort of young people with diabetes in Northern Australia, the prevalence of diabetes-related complications was higher among those with T2D than T1D.

## 1. Introduction

Type 2 diabetes (T2D) in childhood and adolescence is emerging as an important public health challenge worldwide. This condition appears to be rapidly progressive and associated with substantial comorbidities, posing a high risk of early morbidity and mortality [1]. There is a growing body of evidence from the USA and Canada regarding complications of youth-onset T2D [2, 3, and 4]. However, less is known about diabetes complications in youth-onset T2D in northern Australia, despite a high prevalence in this region, arguably the highest in the world within recent years [5].

Youth-onset T2D is a complex, chronic disease, which results from genetic, epigenetic, environmental, and metabolic causes [6]. Higher incidence of youth-onset T2D is associated with a range of demographic and socio-economic risk factors including female sex [3, 7, 8], First Nations ethnicity [7, 8], non-Caucasian descent [3, 9, 10], overweight/obesity [7, 9, 11, 12], rurality [11, 13], and poverty [3, 10, 13]. Family history of diabetes, including maternal diabetes in pregnancy, is common among diagnosed youth [1, 10, 13, 14, and 15], emphasizing the inter-generational cycle of metabolic disease.

There is limited international prevalence and incidence data for youth-onset T2D [16]. High prevalence (≥20 per 100,000 population) has been identified in countries including the USA, Brazil, China, and Kuwait [16]. High incidence of youth-onset T2D has been described in China and India [17]. Longitudinal data demonstrate a clear trend towards increasing incidence of youth-onset T2D globally over recent decades [1, 18].

In Australia, First Nations young people have been identified as particularly high risk. The crude prevalence of youth-onset T2D among First Nations people in northern Australia aged 24 years or younger was 6.7 per 1,000 people [5]. While the incidence has been rising among all Australian young people, the mean incidence observed in First Nations children compared with non-Indigenous children is up to 20-fold higher [7].

T2D in young people has been described as a “severe progressive phenotype” [1]. A high proportion of young people with T2D will have dyslipidaemia and hypertension even at diagnosis and the majority within 15 years [2], which in turn increases the risk of vascular complications. In comparisons of youth with T2D vs. T1D, the prevalence of microvascular complications is higher among youth with T2D despite similar time frames post-diagnosis, glycaemia, and age [19]. Macrovascular complications such as ischaemic heart disease (IHD) and stroke are also more prevalent among cohorts followed post-diagnosis with youth-onset T2D [20]. Mortality is increased, with higher risk of death in those with T2D compared to T1D, even when duration of diabetes is shorter [20]. The reason for the high incidence of complications in youth-onset T2D is unknown but may be related to the extreme metabolic phenotype (including severe insulin resistance and rapid worsening of beta cell function) and challenging socio-economic circumstances [2].

While clinicians are familiar with typical T1D and adult-onset T2D, they may not be aware of the alarmingly high rates of life-threatening vascular complications described in youth-onset T2D. The heterogeneity in phenotypes and uncertainty regarding the course of youth-onset T2D necessitates investigation of patient characteristics and complications, particularly in areas of high disease burden. Establishing complication rates among a large cohort of young people with T2D in northern Australia will highlight the importance of this emerging condition to communities and health systems and help avoid therapeutic inertia.

The aim of this study was, firstly, to assess morbidity among young people with diabetes presenting to a regional hospital in northern Australia and, secondly, to compare the prevalence of complications among those living with T2D versus T1D.

## 2. Methods

This cross-sectional study was a retrospective review of all young people with diabetes who required inpatient management at a regional Northern Australian hospital, between 2015 and 2019. Eligible young people were diagnosed with T1D or T2D between the ages of 1 and 25 years and were admitted to hospital for any reason between 1 January 2015 and 31 December 2019. Age at the time of admission was up to 30 years. Eligible youth were identified using International Classification of Diseases (ICD) codes recorded during the admission. All ICD codes relating to T1D or T2D were used (E10, E11, and other codes related to complications or associations of diabetes). Exclusion criteria included diagnosis with other types of diabetes (such as gestational diabetes, monogenic diabetes, or cystic fibrosis-related diabetes). The allocation of included youth to either the T1D or T2D group was performed solely on the basis of the ICD-10 coding, not by further analysing any clinical or laboratory data.

Individual electronic medical records including inpatient notes, correspondence, and laboratory results were reviewed. The most recent hospital admission within the study period was used as the date of data collection. Data included demographics, anthropometry, diabetes duration, smoking status, blood pressure (BP), medications, biochemistry (HbA1c, lipids, platelets, renal and liver function tests, and urine albumin/creatinine ratio), diabetes complications as recorded in the patient medical record, and mortality. All data were de-identified for analysis after cross-checking for duplication.

Remoteness area index was calculated using the Accessibility/Remoteness Index of Australia (ARIA) score used by the Australian Bureau of Statistics, with ARIA codes Remote (3) and Very Remote (4) considered as “remote” in our data [21]. For those age <18 years, creatinine was recorded in *µ*mol/L. For those age >18 years, eGFR was recorded in mL/min. Albuminuria was defined as urine albumin/creatinine ratio of >3 mg/mmol on a spot urine sample. End-stage renal failure (ESRF) was defined as a requirement for long-term renal replacement therapy. Non-alcoholic fatty liver disease (NAFLD) scores were calculated using the formula described by Angulo et al., with scores above +0.675 considered “probable NAFLD” and scores above −1.455 considered “possible NAFLD” [22]. The components of the NAFLD score are age, body mass index (BMI), hyperglycaemia, AST/ALT ratio, and albumin and platelet count [22]. Major amputation was defined as any lower limb amputation at or above the ankle joint and minor amputation as any lower limb amputation below the ankle joint. Peripheral arterial disease (PAD) was defined as any history of lower limb revascularization, ischaemic limb, or imaging-documented peripheral arterial disease. IHD was defined as any history of myocardial infarction or angina or evidence of ischaemia documented on stress testing. Stroke was defined as any clinical history of stroke, including both ischaemic and haemorrhagic stroke. Retinopathy was defined as any history of retinopathy identified on examination or optometry report; if retinopathy was present, then any history of requiring treatment was recorded. Neuropathy was defined as peripheral neuropathy documented from physical examination, podiatry report, or nerve conduction studies. Pregnancy loss was defined as any miscarriage or pregnancy loss of a confirmed pregnancy at any gestation. Inpatient days per year were calculated by dividing the cumulative length of stay for all admissions by the number of years the patient had the first admission recorded until the end of the study period.

Demographic characteristics, metabolic risk factors, and diabetes complications were compared between young people with T1D and T2D using a *χ*^2^ test or Fisher's exact test (categorical variables), independent *t*-test, or Wilcoxon rank sum test if non-parametric (continuous variables). Continuous variables are given as mean (SD) if normally distributed or median (IQ range). Unadjusted odds ratios were calculated using logistic regression, to describe the likelihood of risk factors and diabetes complications with a diagnosis of T2D, compared to T1D. Multivariable logistic regression was used for those diabetes complications with *n* > 20, to calculate adjusted odds ratios, adjusting for age, sex, BMI, smoking, HbA1c, and Aboriginal and/or Torres Strait Islander ethnicity. Statistical analysis was performed using Stata 15.1 (StataCorp, Texas, USA).

Ethical approval for this study was granted by the Far North Queensland Human Research Ethics Committee (Reference HREC/2019/QCH/53795–1379). A waiver of consent was granted. Following consultation, support was given by the following organizations: Wuchopperen Health Service, Apunipima Cape York Health Council, Aboriginal and Torres Strait Islander Health (Torres and Cape Hospital and Health Service), and Cairns Diabetes Centre (Cairns and Hinterland Hospital and Health Service).

## 3. Results

A total of 357 young people with diabetes met the inclusion criteria. This included 165 youth with T1D and 192 youth with T2D. An additional 103 potential youth identified through ICD codes were excluded after review of individual medical records due to absence of confirmed diabetes diagnosis, gestational diabetes, cystic fibrosis, or monogenic diabetes. Demographic characteristics at the time of the most recent admission are shown in Table 1. Youth with T2D were diagnosed at an older age (mean age 18 vs. 13 years, *p*  < 0.001) *and had a shorter diabetes duration* (*p* =0.012) compared with youth with T1D. Youth with T2D were more likely to be female, live remotely, and identify as Aboriginal and/or Torres Strait Islander compared with youth with T1D.

Young people with T2D had a lower median HbA1c than those with T1D (Table 1). They also differed from those with T1D in terms of other cardiometabolic characteristics, having higher mean BMI, higher prevalence of smoking, higher mean systolic and diastolic BP, higher mean triglyceride concentration, and lower HDL concentration. Among young people with T2D, 52% were prescribed insulin therapy, 85% metformin, and 10%–15% other diabetes medications. More youth with T2D were prescribed anti-hypertensives and dyslipidaemic medication than those with T1D.

**Figure 1:**
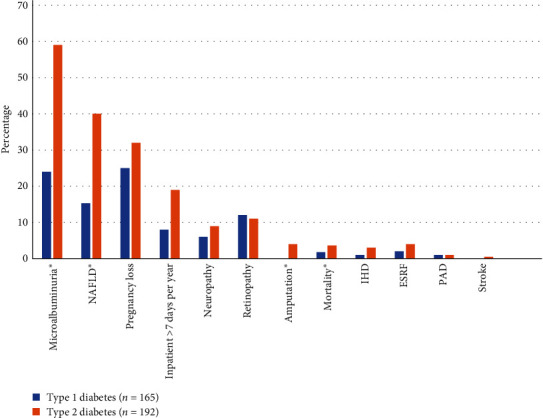
Prevalence of complications in youth with type 1 and type 2 diabetes.  ^*∗*^indicates a statistically significant difference between T1D and T2D cohorts. Non-alcoholic fatty liver disease (“NAFLD”) in this figure refers to either “possible” or “probable” NAFLD, based on NAFLD score. Total *n* is lower for some complications due to missing data: urine microalbumin, *n* = 174; NAFLD score, *n* = 260. Total *n* is lower for pregnancy loss, as these percentages refer only to women with a history of known pregnancy, *n* = 89.

Diabetes-related comorbidities are shown in Figure 1 and Table S1. Despite a lower median HbA1c and shorter mean diabetes duration, several diabetes complications were more prevalent among young people with T2D versus T1D, including microalbuminuria (59% vs. 24%, *p*  < 0.001), elevated NAFLD score consistent with either “possible” or “probable” NAFLD (40% vs. 15%, *p*  < 0.001), and amputation (4% vs. 0%, *p*=0.008). Young people with T2D had a higher median number of inpatient days per year (2.75 vs. 1.0, *p*  < 0.00). There was no significant difference by diabetes type for ESRF (4% vs. 2%, *p*=0.285), neuropathy (9% vs. 6%, *p*=0.284), retinopathy (7% vs. 7%, *p*=0.871), IHD (3% vs. 1%, *p*=0.218), stroke (0.5% vs. 0%, *p*=0.348), or pregnancy loss (among young women who had at least one recorded pregnancy, 32% vs. 25%, *p*=0.71). There was no significant difference in mortality over the 5-year study duration between those with T2D and T1D in this young cohort (3.6% vs. 1.8%, *p*=0.297).

ESRF and major amputation had a large impact on inpatient days per year in a small number of individuals with very prolonged admissions (data not shown). Of the 10 young people with ESRF, seven had T2D, and eight were Aboriginal or Torres Strait Islander, and the mean diabetes duration was 15 years (range 8–25 years, SD 5.6 years). Among the eight young people with amputations, all eight had T2D, and six were Aboriginal or Torres Strait Islander, with a mean diabetes duration 11 years (range 3–18 years, SD 4.9). Among young Aboriginal and Torres Strait Islander people (with either T1D or T2D), there was a significantly increased risk of microalbuminuria, elevated NAFLD score, and extended inpatient stay compared to non-Indigenous youth (58% vs. 22%, 32% vs. 20%, 21% vs. 8% respectively, *p*  < 0.05). Among the subgroup of 95 young people under age 18 (Table S2), there were higher rates of obesity, anti-hypertensive use, and microalbuminuria in T2D compared to T1D. In this younger group, there were no recorded cases of ESRF, neuropathy, retinopathy, peripheral arterial disease, IHD, stroke, or mortality.

Unadjusted and adjusted odds ratios are given for metabolic characteristics and diabetes complications comparing those with T2D and T1D in Table 2. Model 1 was adjusted for age and sex. Model 2 was adjusted for age, sex, and BMI. Model 3 was adjusted for age, sex, and smoking status. Model 4 was adjusted for age, sex, and Aboriginal and/or Torres Strait Islander ethnicity. Odds ratios were similar to Model 1 when adjusting for age, sex, and HbA1c (data not shown). Duration of diabetes was not adjusted for, due to inadequate sample size secondary to limited information available. There was a significant correlation between Aboriginal and Torres Strait Islander ethnicity and variables including remoteness, smoking, and inpatient days per year (all *p*  < 0.01).

Unadjusted OR were significantly higher for young people with T2D compared to T1D for prevalence of smoking, remote residence, BMI >30 kg/m^2^, high systolic BP, high diastolic BP, low HDL, high triglycerides and for rates of microalbuminuria, positive NAFLD score, and higher number of inpatient days per year. When adjusted for age and sex, the difference in blood pressure was no longer significantly different between groups, but other differences remained. When adjusted for age, sex, and smoking (Model 3) and age, sex, and Aboriginal or Torres Strait Islander ethnicity (Model 4), there was an increased OR for low HDL, positive NAFLD score, and microalbuminuria but no difference in triglycerides and number of inpatient days per year with T2D compared to T1D, in both models. When adjusted for age, sex, and BMI, the OR was no longer significantly different for low HDL, high triglycerides, or positive NAFLD score, indicating BMI was a greater contributor to those comorbidities than the type of diabetes. A significantly increased risk of microalbuminuria remained in all models, with T2D compared to T1D.

## 4. Discussion

In this cohort of 357 young people living with diabetes and requiring inpatient care at a northern Australian hospital, we identified significant morbidity from T2D complications and hospitalizations. The major findings among young people living with T2D, compared to those living with T1D, included a significantly higher prevalence of cardiometabolic comorbidities, diabetes-related comorbidities (microalbuminuria, NALFD, and amputation), and more inpatient days per year.

In this cohort, several adverse cardiometabolic characteristics were more prevalent among those with T2D compared to those with T1D. The strong association of youth-onset T2D with cardiometabolic risk factors is concerning for the future risk of cardiovascular conditions. In our cohort, those with T2D had a higher rate of obesity, smoking prevalence, mean systolic and diastolic BP, mean triglycerides, and NAFLD score, and lower HDL, than youth with T1D. The greater prevalence of hypertension among those with youth-onset T2D is consistent with other Australian [23, 24] and international [25] studies, though, in this cohort, the difference did not persist following adjustment for age and sex. The high prevalence of dyslipidaemia (specifically elevated triglycerides and low HDL) has also been found in other studies of youth-onset T2D [26]. Given that the OR adjusted for age, sex, and BMI no longer found a difference between groups for these lipid abnormalities, it is likely that obesity was a significant contributor to dyslipidaemia in this cohort. We also found a very high prevalence of smoking, with 46% of those with T2D being current smokers, compared to 17% of adults in North Queensland [27]. While overt IHD was uncommon (3%) in our T2D cohort, noting a mean diabetes duration of only 5.8 years, an Australian study with a mean 12-year diabetes duration indicated a 14% prevalence of IHD/stroke among those with youth-onset T2D, substantially higher than in those with T1D [20].

Among this young cohort, the complications of microalbuminuria and amputation were more prevalent among those with T2D versus T1D, despite a lower median HbA1c and shorter mean diabetes duration. Internationally, in comparisons of youth living with T2D vs. T1D, the prevalence of these microvascular complications is higher among those with T2D despite similar disease duration, glycaemia, and age [4, 19]. Renal disease is a common and early complication of youth-onset T2D, with a higher risk of progression than in youth-onset T1D or later-onset T2D [28]. Our northern Australian cohort data are consistent with this, with a three- to fivefold increased risk of microalbuminuria in youth with T2D compared to T1D, even when adjusting for age, sex, BMI, and Aboriginal and/or Torres Strait Islander ethnicity. In some published cohorts, the prevalence of neuropathy has been higher in T2D compared to T1D [4], while in others, the rates are similar [23], though the T2D cohorts had shorter diabetes duration. Alarmingly, in our cohort, 4% of youth with T2D had undergone an amputation, compared with no youth with T1D. Concerningly, a pregnancy loss occurred in 25%–30% of young women with any history of known pregnancy in both T1D and T2D groups, with a non-statistically significant higher prevalence of pregnancy loss among the T2D group. Population data suggest pregnancy loss (miscarriage or stillbirth) occurs in 19% of pregnancies among Australian women age 18–27 years [29]. Among women with diabetes, higher stillbirth and neonatal death rates have been observed in T2D compared to T1D [30].

In this cohort, the group with T2D experienced more inpatient days per year, with potential consequent impact on quality of life. Youth-onset T2D substantially impacts on quality of life for many young people [31]. In our cohort, those with T2D had a higher median number of inpatient days per year, with 19% spending >7 days per year admitted to hospital. A high burden of hospitalization occurred in Aboriginal or Torres Strait Islander youth. This is consistent with national data demonstrating that hospital admission for T2D is 10 times more likely among First Nations adolescents than non-First Nations adolescents [32]. Aboriginal and Torres Strait Islander ethnicity in this context likely represents greater unmeasured socio-economic inequities and inter-generational effects of historical experience and marginalization on metabolic health [33].

A strength of this study is the use of hospital records to identify all patients with youth-onset diabetes requiring inpatient care rather than limiting the data collection to those who may opt to participate. This is more likely to capture those with low healthcare engagement and psychosocial barriers to accessing routine diabetes care and may reduce selection bias.

There were a number of limitations to this study. Firstly, the retrospective nature of the data collection leads to some incompleteness of data, as some patient characteristics and diabetes complications may not be recorded in a complete manner in the clinical record. The outcome of “possible” or “probable” NAFLD was assessed based on the screening NAFLD score calculator using biochemical, age, and BMI data rather than elastography. Secondly, data may have been inaccurate with respect to diabetes type in some cases, as classification was based on medical records with incomplete pathology data to assist in verification. Thirdly, this cohort may represent a higher disease severity due to the requirement for a hospital presentation for inclusion in the dataset. Patients with youth-onset diabetes who do not require inpatient care may have fewer cardiometabolic risk factors and diabetes-related complications. Fourthly, this dataset did not contain any socio-economic data as this is not routinely recorded during clinical encounters. Therefore, this study did not explore the link between socio-economic factors and diabetes complications, which is likely to be an important component of the risk profile.

These findings imply a number of priorities for communities, clinicians, and health systems. Firstly, the heavy health burden of youth-onset T2D in northern Australia calls for urgent strategies to address social and environmental determinants of health and improve access to evidence-based and culturally appropriate care, to optimize outcomes for young people with T2D. This includes resourcing for smoking cessation strategies, given the high prevalence of smoking identified among young people with diabetes. Secondly, there is a clear need for vigilance in detecting T2D and addressing comorbidities as early as possible, given the very high prevalence of diabetes-specific complications at just 5.8 years mean disease duration, and the occurrence of major end-organ complications (ESRF and amputation) in some young people at less than 10 years of diabetes duration. Admission to hospital is an opportune time to screen for complications and plan appropriate management strategies moving forward; however, ideally hospital admission could be avoided if young people were linked in with holistic and multidisciplinary primary care teams. Thirdly, management strategies that are acceptable and feasible for northern Australian communities must be developed to address the complications and quality-of-life implications of youth-onset T2D. Dedicated models of care co-designed with young people and their communities are required to reduce diabetes stigma, broaden social support. and consider the delivery of health services in youth-friendly environments [34].

## 5. Conclusions

In a cohort of young people with diabetes accessing care at a northern Australian hospital, the prevalence of cardiometabolic comorbidities and diabetes-related complications was higher among those with T2D than those with T1D. Young people with T2D had micro-albuminuria detected very early and experienced a high burden of inpatient care days. Understanding the devastating complications and burden of disease experienced by youth-onset T2D patients in Northern Australia is important for developing solutions. New approaches to addressing upstream social determinants of metabolic health, as well as preventing and managing T2D in young people, are urgently required, including hearing the voices of young people and communities.

## Figures and Tables

**Table 1:** Patient characteristics, according to type of diabetes.

Patient characteristic	Total (*n* = 357)	Type 1 diabetes (*n* = 165 (46%))	Type 2 diabetes (*n* = 192 (54%))	*p*
*Demographics*				
Age at admission, mean (SD), y	22 (5.9)	**20 (5.6)**	**24 (5.5)**	**<0.001**
Age at diagnosis, mean (SD), y	15.4 (6.2)	**13 (5.7)**	**18 (5.4)**	**<0.001**
Diabetes duration, mean (SD), y	6.7 (6)	**7.6 (6.6)**	**5.8 (5.3)**	**0.012**
Duration of diabetes				
<5 year, *n* (%)	141 (40%)	63 (38%)	78 (40%)	0.05
5–10 year, *n* (%)	84 (23%)	44 (26%)	40 (21%)	
>10 year, *n* (%)	77 (22%)	45 (27%)	32 (17%)	
Unknown duration, *n* (%)	55 (15%)	13 (8%)	42 (22%)	
Male sex, *n* (%)	132 (39%)	**80 (48%)**	**60 (31%)**	**0.001**
Ethnicity				
Non-Indigenous	173 (48%)	**136 (82%)**	**32 (17%)**	**<0.001**
Aboriginal	105 (29%)	**21 (13%)**	**84 (44%)**	—
Torres Strait Islander	48 (13%)	**4 (2%)**	**44 (23%)**	—
Both Aboriginal and TSI	37 (11%)	**4 (2%)**	**32 (17%)**	—
Remote or very remote postcode (ARIA 3 or 4)	100 (28%)	**24 (15%)**	**75 (39%)**	**<0.001**
*Cardiometabolic characteristics*				
HbA1c, median (IQR)				
(%)	8.8 (7.1–11.2)	**9.2 (7.9–11.2)**	**8.5 (6.7–11.4)**	**0.018**
(mmol/mol)	73 (54–99)	**77 (63–99)**	**69 (50–101)**	—
HbA1c >9% (>75 mmol/mol)	160 (40%)	83 (55%)	77 (44%)	0.054
Smoking, current *n* (%)	101 (33%)	**28 (20%)**	**73 (46%)**	**<0.001**
BMI	28 (8.0)	**24 (5.6)**	**32 (7.7)**	**<0.001**
BMI ≥ 30	93 (34%)	**16 (12%)**	**77 (55%)**	**<0.001**
Systolic BP, mean (SD)	122 (17)	**120 (16)**	**124 (18)**	**0.034**
Systolic BP >130, *n* (%)	82 (25%)	**28 (18%)**	**54 (30%)**	**0.023**
Diastolic BP, mean (SD)	76 (12)	**74 (12)**	**77 (11)**	**0.004**
Diastolic BP >80, *n* (%)	107 (32%)	**35 (23%)**	**72 (40%)**	**0.002**
Total cholesterol, mmol/L, median (IQR)	5.0(4.1–5.8)	5.0 (4.5–6.0)	4.9 (4.0–5.7)	0.312
Total cholesterol >4 mmol/L, *n* (%)	98 (77%)	28 (87%)	69 (74%)	0.119
LDL, mmol/L,mean (SD)	2.8 (0.98)	3.1 (0.95)	2.7 (0.98)	0.09
LDL >2.5 mmol/L, *n* (%)	67 (60%)	20 (69%)	46 (58%)	0.27
HDL, mmol/L,median (IQR)	1.0(1.9–1.2)	**1.26 (1.0–1.5)**	**1.0 (0.8–1.1)**	**<0.001**
HDL <1 mmol/L, *n* (%)	49 (41%)	**6 (20%)**	**43 (49%)**	**0.006**
Triglycerides, mmol/L, med (IQR)	2.1(1.3–2.4)	**1.0 (0.9–2.6)**	**2.4 (1.6–3.8)**	**<0.001**
Triglycerides >2.0 mmol/L, *n* (%)	62 (50%)	**9 (30%)**	**53 (58%)**	**0.009**
*Medication use*				
Insulin, *n* (%)	263 (74%)	**165 (100%)**	**98 (52%)**	**<0.001**
Metformin, *n* (%)	167 (47%)	**3 (5%)**	**162 (85%)**	**<0.001**
SGLT2i	30 (8%)	**1 (0.6%)**	**29 (15%)**	**<0.001**
Sulfonylurea	18 (5%)	**0**	**18 (10%)**	**<0.001**
DPP-4i	20 (6%)	**1 (0.6%)**	**19 (10%)**	**<0.001**
GLP-1a	22 (6%)	**0**	**22 (12%)**	**<0.001**
Anti-hypertensive therapy	57 (16%)	**4 (2%)**	**53 (28%)**	**<0.001**
Lipid-lowering therapy	31 (9%)	**3 (2%)**	**28 (15%)**	**<0.001**

ARIA, accessibility/remoteness index of Australia; BMI, body mass index; BP, blood pressure; DPP4i, dipeptidyl peptidase IV inhibitor; GLP1a, glucagon-like peptide receptor agonist; HDL, high-density lipoprotein; LDL, low-density lipoprotein; SGLT2i, sodium glucose co-transport 2 inhibitor; TSI, Torres Strait Islander. Total *n* is lower for some characteristics due to missing data: diabetes duration, *n* = 302; HbA1c, *n* = 331; BMI, *n* = 271; BP, *n* = 337; total cholesterol, *n* = 127; LDL, *n* = 109; HDL, *n* = 118; Trig, *n* = 124. The values in bold are associated with a *p*-value <0.05, representing a significant difference between youth with Type 1 diabetes and youth with Type 2 diabetes.

**Table 2:** Adjusted odds ratios of patient characteristics and comorbidities with T2D, compared to T1D.

Characteristics/comorbidities	Unadjusted OR (95% CI)	*p*	Model 1 OR (95% CI) adjusted for age and sex	*p*	Model 2 OR (95% CI) adjusted for age, sex and BMI	*p*	Model 3 OR (95% CI) adjusted for age, sex, and smoking	*p*	Model 4 OR (95% CI) adjusted for age, sex, and Aboriginal and/or TSI ethnicity	*p*
HbA1c >9% (>75 mmol/mol)	0.65 (0.41–1.00)	0.054	0.88 (0.54–1.43)	0.62	1.02 (0.53–1.94)	0.95	0.96 (0.56–1.60)	0.91	0.63 (0.33–1.20)	0.16
Smoking	**3.01 (1.8–4.9)**	**<0.001**	**2.0 (1.17–3.45)**	**0.01**	**2.52 (1.20–5.28)**	**0.01**	—		1.13 (0.56–2.30)	0.71
Remote location	**3.76 (2.23–6.34)**	**<0.001**	**3.84 (2.12–6.75)**	**<0.001**	**4.75 (2.27–9.95)**	**<0.001**	**3.82 (2.02–7.21)**	**<0.001**	1.33 (0.65–2.69)	0.42
Aboriginal or TSI ethnicity	**23.5 (13.5–40.7)**	**<0.001**	**25.4 (13.7–47.5)**	**<0.001**	**24.2 (10.5–55.5)**	**<0.001**	**27.1 (13.3–55.2)**	**<0.001**	—	—
BMI ≥30 kg/m^2^	**8.91 (4.7–16.4)**	**<0.001**	**6.9 (3.6–13.3)**	**<0.001**	—	—	**6.41 (3.15–13.4)**	**<0.001**	**8.5 (3.77–19.1)**	**<0.001**
Systolic BP >130 mmHg	**1.86 (1.08–3.06)**	**0.02**	1.48 (0.84–2.62)	0.17	1.14 (0.54–2.78)	0.72	1.59 (0.85–2.97)	0.14	0.99 (0.48–2.1)	0.98
Diastolic BP >80 mmHg	**2.13 (1.31–3.45)**	**0.002**	1.49 (0.87–2.54)	0.14	1.10 (0.55–2.19)	0.77	1.44 (0.80–2.58)	0.24	0.90 (0.44–1.84)	0.79
Total cholesterol >4 mmol/L	0.41 (0.13–1.29)	0.12	0.48 (0.14–1.58)	0.23	0.67 (0.17–2.65)	0.57	0.51 (0.15–1.75)	0.29	0.52 (0.13–2.05)	0.35
LDL >2.5 mmol/L	0.61 (0.24–1.50)	0.28	0.64 (0.25–1.64)	0.35	0.64 (0.19–2.0)	0.44	0.77 (0.28–2.13)	0.62	0.42 (0.12–1.44)	0.17
HDL<1.0 mmol/L	**3.81 (1.42–10.2)**	**0.008**	**3.72 (1.34–10.30)**	**0.01**	3.00 (0.84–10.6)	0.08	**3.75 (1.14–9.87)**	**0.01**	2.97 (0.93–9.44)	0.06
Triglycerides >2.0 mmol/L	**3.17 (1.31–7.67)**	**0.01**	**2.89 (1.15–7.29)**	**0.02**	2.33 (0.70–7.75)	0.16	2.27 (0.85–6.05)	0.09	2.21 (0.75–6.50)	0.14
Microalbuminuria ≥3 mg/mmol	**4.65 (2.29–9.41)**	**<0.001**	**4.5 (2.2–9.4)**	**<0.001**	**4.84 (1.83–12.8)**	**0.001**	**6.77 (2.91–15.8)**	**<0.001**	**2.58 (1.04–6.38)**	**0.04**
Possible NAFLD >−1.455	**3.37 (1.82–6.21)**	**<0.001**	**2.28 (1.19–4.40)**	**0.01**	1.05 (0.47–2.31)	0.90	**2.64 (1.31–5.34)**	**0.007**	**2.61 (1.17–5.81)**	**0.01**
Neuropathy	1.58 (0.67–3.68)	0.28	0.81 (0.33–1.99)	0.65	1.31 (0.39–4.33)	0.65	0.72 (0.26–1.95)	0.52	0.60 (0.19–1.92)	0.39
Retinopathy	0.93 (0.41–2.11)	0.87	0.45 (0.19–1.08)	0.07	0.85 (0.27–3.65)	0.76	0.45 (0.17–1.17)	0.10	0.32 (0.09–1.06)	0.06
Pregnancy loss	1.48 (0.46–4.73)	0.50	1.34 (0.41–4.32)	0.62	0.75 (0.14–3.89)	0.73	1.89 (0.42–8.40)	0.40	0.93 (0.16–5.27)	0.94
Inpatient days per year >7 days	**2.57 (1.33–4.95)**	**0.005**	1.83 (0.91–3.68)	0.08	**1.05 (1.01–1.09)**	**0.015**	1.99 (0.98–4.03)	0.05	0.91 (0.37–2.21)	0.84

BMI, body mass index; BP, blood pressure; HDL, high-density lipoprotein; LDL, low-density lipoprotein; NAFLD, non-alcoholic fatty liver disease; TSI, Torres Strait Islander. The values in bold are the odds ratios associated with a *p*-value <0.05.

## Data Availability

Data are available on reasonable request from the corresponding author Dr Kate Hawke, kate.hawke@uqconnect.edu.au.

## Supplementary Materials

Supplementary Materials Table S1: diabetes-related comorbidities, according to type of diabetes. Table S2: patient characteristics, according to type of diabetes in young people aged <18 years.

## References

[1] Viner R., White B., Christie D. (2017). Type 2 diabetes in adolescents: a severe phenotype posing major clinical challenges and public health burden. *The Lancet*.

[2] Bjornstad P., Drews K. L., Caprio S. (2021). Long-term complications in youth-onset type 2 diabetes. *New England Journal of Medicine*.

[3] Dabelea D., Stafford J. M., Mayer-Davis E. J. (2017). Association of type 1 diabetes vs type 2 diabetes diagnosed during childhood and adolescence with complications during teenage years and young adulthood. *JAMA*.

[4] Dart A. B., Martens P. J., Rigatto C., Brownell M. D., Dean H. J., Sellers E. A. (2014). Earlier onset of complications in youth with type 2 diabetes. *Diabetes Care*.

[5] Titmuss A., Davis E. A., O’Donnell V. (2022). Youth-onset type 2 diabetes among First Nations young people in northern Australia: a retrospective, cross-sectional study. *The Lancet Diabetes & Endocrinology*.

[6] Misra S., Ke C., Srinivasan S. (2023). Current insights and emerging trends in early-onset type 2 diabetes. *The Lancet Diabetes & Endocrinology*.

[7] Haynes A., Kalic R., Cooper M., Hewitt J. K., Davis E. A. (2016). Increasing incidence of type 2 diabetes in Indigenous and non-Indigenous children in Western Australia, 1990–2012. *Medical Journal of Australia*.

[8] Stone M., Baker A., Maple Brown L. (2013). Diabetes in young people in the top end of the northern territory. *Journal of Paediatrics and Child Health*.

[9] Scott A., Toomath R., Bouchier D. (2006). First National audit of the outcomes of care in young people with diabetes in New Zealand: high prevalence of nephropathy in Maori and Pacific Islanders. *New Zealand Medical Journal*.

[10] Copeland K. C., Zeitler P., Geffner M. (2011). Characteristics of adolescents and youth with recent-onset type 2 diabetes: the TODAY cohort at baseline. *The Journal of Clinical Endocrinology & Metabolism*.

[11] Craig M. E., Femia G., Broyda V., Lloyd M., Howard N. J. (2007). Type 2 diabetes in Indigenous and non-Indigenous children and adolescents in New South Wales. *Medical Journal of Australia*.

[12] Wheelock K. M., Sinha M., Knowler W. C., Nelson R. G., Fufaa G. D., Hanson R. L. (2016). Metabolic risk factors and type 2 diabetes incidence in American Indian children. *The Journal of Clinical Endocrinology & Metabolism*.

[13] Dart A. B., Sellers E. A., Martens P. J., Rigatto C., Brownell M. D., Dean H. J. (2012). High burden of kidney disease in youth-onset type 2 diabetes. *Diabetes Care*.

[14] Dabelea D., Hanson R. L., Lindsay R. S. (2000). Intrauterine exposure to diabetes conveys risks for type 2 diabetes and obesity: a study of discordant sibships. *Diabetes*.

[15] Mendelson M., Cloutier J., Spence L., Sellers E., Taback S., Dean H. (2011). Obesity and type 2 diabetes mellitus in a birth cohort of First Nation children born to mothers with pediatric-onset type 2 diabetes. *Pediatric Diabetes*.

[16] Lynch J. L., Barrientos-Pérez M., Hafez M. (2021). Country-specific prevalence and incidence of youth-onset type 2 diabetes: a narrative literature review. *Annals of Nutrition and Metabolism*.

[17] Wu H., Patterson C. C., Zhang X. (2022). Worldwide estimates of incidence of type 2 diabetes in children and adolescents in 2021. *Diabetes Research and Clinical Practice*.

[18] Lawrence J. M., Divers J., Isom S. (2021). Trends in prevalence of type 1 and type 2 diabetes in children and adolescents in the US, 2001–2017. *JAMA*.

[19] Perng W., Conway R., Mayer-Davis E., Dabelea D. (2023). Youth-onset type 2 diabetes: the epidemiology of an awakening epidemic. *Diabetes Care*.

[20] Constantino M. I., Molyneaux L., Limacher-Gisler F. (2013). Long-term complications and mortality in young-onset diabetes: type 2 diabetes is more hazardous and lethal than type 1 diabetes. *Diabetes Care*.

[21] Australian Bureau of Statistics (2023). Remoteness structure—Australian statistical geography standard 2021. https://www.abs.gov.au/statistics/standards/australian-statistical-geography-standard-asgs-edition-3/jul2021-jun2026/remoteness-structure.

[22] Angulo P., Hui J. M., Marchesini G. (2007). The NAFLD fibrosis score: a noninvasive system that identifies liver fibrosis in patients with NAFLD. *Hepatology*.

[23] Eppens M. C., Craig M. E., Cusumano J. (2006). Prevalence of diabetes complications in adolescents with type 2 compared with type 1 diabetes. *Diabetes Care*.

[24] Titmuss A., Davis E. A., Brown A., Maple-Brown L. J. (2019). Emerging diabetes and metabolic conditions among Aboriginal and Torres Strait Islander young people. *Medical Journal of Australia*.

[25] Amutha A., Mohan V. (2016). Diabetes complications in childhood and adolescent onset type 2 diabetes—a review. *Journal of Diabetes and its Complications*.

[26] Amutha A., Datta M., Unnikrishnan R., Anjana R. M., Mohan V. (2012). Clinical profile and complications of childhood- and adolescent-onset type 2 diabetes seen at a diabetes center in south India. *Diabetes Technology & Therapeutics*.

[27] AIHW (2024). Australian Institute of Health and Welfare Data tables: for Primary Health Network areas—current daily smokers and alcohol consumption 2017–18. https://www.aihw.gov.au/reports/alcohol/alcohol-tobacco-other-drugs-australia/data.

[28] Bjornstad P., Cherney D. Z., Maahs D. M., Nadeau K. J. (2016). Diabetic kidney disease in adolescents with type 2 diabetes: new insights and potential therapies. *Current Diabetes Reports*.

[29] Herbert D., Lucke J., Dobson A. (2009). Pregnancy losses in young Australian women: findings from the Australian longitudinal study on women’s health. *Women’s Health Issues*.

[30] Murphy H. R., Howgate C., O’Keefe J. (2021). Characteristics and outcomes of pregnant women with type 1 or type 2 diabetes: a 5-year national population-based cohort study. *The Lancet Diabetes & Endocrinology*.

[31] Copeland K. C., Silverstein J., Moore K. R. (2013). Management of newly diagnosed type 2 diabetes mellitus (T2DM) in children and adolescents. *Pediatrics*.

[32] Azzopardi P. S., Sawyer S. M., Carlin J. B. (2018). Health and wellbeing of Indigenous adolescents in Australia: a systematic synthesis of population data. *The Lancet*.

[33] Australian Institute of Health and Welfare (2022). Determinants of health for Indigenous Australians. https://www.aihw.gov.au/reports/australias-health/social-determinants-and-indigenous-health.

[34] Weaver E., Freeman N., Mack S. I don’t really know what diabetes is: a qualitative study exploring the experiences of Aboriginal and Torres Strait Islander young people aged 10 to 25 years living with type 2 diabetes in Northern and Central Australia. *Candian Journal of Diabetes*.

