# Supplementary material for: Diabetes Complications among Inpatients with Childhood and Young Adult–Onset Type 1 and 2 Diabetes
Source: Pediatr Diabetes. 2024 Jun 14;2024:9926090. doi: 10.1155/2024/9926090 (PMC12016798; doi:10.1155/2024/9926090)
Supplement: Supplementary Materials — Table S1: diabetes-related comorbidities, according to type of diabetes. Table S2: patient characteristics, according to type of diabetes in young people aged <18 years. [file 9926090.f1.docx]

Supplementary Table 1

**Table S1: Diabetes-related comorbidities, according to type of diabetes**

|  | **Total**  **N=357** | **Type 1 diabetes**  **n=165 (46%)** | **Type 2 diabetes**  **n=192 (54%)** | **p** |
| --- | --- | --- | --- | --- |
| Urine ACR mg/mmol, median (IQR) | 2.5 (0.9-15) | **1.3 (0.6-2.5)** | **5.8 (1.3-31)** | **<0.001** |
| Microalbuminuria ≥3mg/mmol n(%) | 82 (47%) | **14 (24%)** | **68 (59%)** | **<0.001** |
| Abnormal GFR <90, n(%) | 36 (14%) | 11 (10%) | 25 (15%) | 0.225 |
| Creatinine umol/l, median (IQR) | 57 (47-70) | 58 (48-71) | 55 (45-67) | 0.425 |
| ESRF, n(%) | 10 (3%) | 3 (2%) | 7 (4%) | 0.285 |
| Albumin, mean (SD) | 37 (8) | **39 (7)** | **36 (7)** | **<0.001** |
| AST, median (IQR) | 19 (13-28) | **16 (12-22)** | **22 (14-32)** | **<0.001** |
| ALT, median (IQR) | 22 (15-37) | **17 (13-24)** | **28 (17-48)** | **<0.001** |
| Platelets, mean (SD) | 285 (78) | 281 (72) | 288 (83) | 0.443 |
| NAFLD score | -2.31 (1.7) | **-2.8 (1.4)** | **-1.8 (1.9)** | **<0.001** |
| Possible NAFLD, n(%)^a^ | 68 (26%) | **18 (15%)** | **49 (37%)** | **<0.001** |
| Probable NALFD, n(%)^b^ | 9 (3.4%) | **1 (0.3%)** | **8 (3%)** | **0.025** |
| Minor amputation, n(%) | 6 (1.7%) | **0** | **6 (3%)** | **0.021** |
| Major amputation, n(%) | 2 (0.5%) | 0 | 2 (1%) | 0.185 |
| Peripheral arterial disease | 4 (1%) | 2 (1%) | 2 (1%) | 0.886 |
| Neuropathy | 25 (7%) | 9 (6%) | 16 (9%) | 0.284 |
| Retinopathy | 25 (7%) | 12 (7%) | 13 (7%) | 0.871 |
| Retinopathy requiring treatment | 11 (5%) | 5 (5%) | 6 (4%) | 0.833 |
| Ischaemic heart disease | 8 (2%) | 2 (1%) | 6 (3%) | 0.218 |
| Stroke | 1 (0.3%) | 0 | 1 (0.5%) | 0.348 |
| Pregnancy loss | 22 (30%) | 4 (25%) | 18 (32%) | 0.711 |
| Mortality, n(%) | 10 (2.8%) | 3 (1.8%) | 7 (3.6%) | 0.297 |
| Inpatient days per year | 2 (0.3-4.0) | **1 (0-3)** | **2.75 (1-6)** | **<0.001** |
| Inpatient >7d per year, n(%) | 51 (14%) | **14 (8%)** | **37 (19%)** | **0.004** |

ACR = Albumin creatinine ratio; ALT = alanine transaminase; AST = aspartate aminotransferase; ESRF = End-stage renal failure; GFR = Glomerular filtration rate; NAFLD = Non-alcoholic fatty liver disease. NALFD scores = a. scores above -1.455 considered ‘possible NAFLD’,

b. scores above +0.675 considered ‘probable NAFLD’.

Total n is lower for some variables: ACR n=174, GFR n=269, Creatinine n=177, ALT n=336, AST n=332, NAFLD score n=260, Platelets n=338, history of known pregnancy n=89.

Supplementary Table 2

**Table S2: Patient characteristics, according to type of diabetes in young people aged <18 years**

| Characteristics of patients aged <18 years | **Total**  **n=95** | **Type 1 diabetes**  **n=64 (67%)** | **Type 2 diabetes**  **n=31 (33%)** | **p** |
| --- | --- | --- | --- | --- |
| ***Demographics*** | | | | |
| Age at admission, mean (SD), y | 14 (2.1) | 14 (2.1) | 14 (2.2) | 0.912 |
| Diabetes duration, mean (SD), y | 3.6 (2.9) | **4.1 (3.6)** | **2.5 (1.6)** | **0.022** |
| Male sex, n(%) | 40 (42%) | **33 (52%)** | **7 (23%)** | **0.007** |
| Ethnicity: Non-Indigenous | 57 (60%) | **51 (80%)** | **6 (19%)** | **<0.001** |
| Remote or very remote postcode (ARIA 3 or 4) | 23 (24%) | **5 (8%)** | **18 (58%)** | **<0.001** |
| ***Cardiometabolic characteristics*** |  |  |  |  |
| HbA1c % (median, IQR) | 9.2 (7.7-11.3) | 9.2 (8.2-11.3) | 9.1 (7.2-12) | 0.712 |
| BMI | 24 (6.0) | **23 (5.3)** | **29 (5.5)** | **<0.001** |
| Systolic BP>130, n(%) | 9 (11%) | 5 (9%) | 4 (15%) | 0.490 |
| Diastolic BP>80, n(%) | 10 (12%) | 6 (11%) | 4 (15%) | 0.677 |
| Antihypertensive therapy, n(%) | 6 (6%) | **0** | **6 (19%)** | **<0.001** |
| Microalbuminuria, n(%) | 14 (41%) | **3 (20%)** | **11 (58%)** | **0.026** |
| NAFLD score positive, n(%) | 9 (13%) | 5 (10%) | 4 (18%) | 0.350 |

ARIA = Accessibility/Remoteness Index of Australia; BMI = Body Mass Index; BP = Blood Pressure. ‘NAFLD’ (Non-Alcoholic Fatty Liver Disease) score positive in this Table refers to either ‘possible’ or ‘probable’ NAFLD, based on NAFLD score.

Total n is less for the following data: HbA1c n=92, BMI n=82, Systolic and diastolic BP n=82, microalbuminuria n=34, NAFLD score n=71.

There were no significant differences between groups with T1D and T2D aged <18 years for the following characteristics and complications: smoking rate, total cholesterol levels, HDL, LDL, triglyceride levels, use of lipid-lowering therapy, glomerular filtration rate, creatinine, inpatient days per year and pregnancy loss.

None of the following complications occurred in this group with either T1D or T2D aged <18 years: retinopathy, neuropathy, end stage renal failure, peripheral arterial disease, amputation, ischemic heart disease, stroke or mortality.
